# Interpretation of Appearance: The Effect of Facial Features on First Impressions and Personality

**DOI:** 10.1371/journal.pone.0107721

**Published:** 2014-09-18

**Authors:** Karin Wolffhechel, Jens Fagertun, Ulrik Plesner Jacobsen, Wiktor Majewski, Astrid Sofie Hemmingsen, Catrine Lohmann Larsen, Sofie Katrine Lorentzen, Hanne Jarmer

**Affiliations:** 1 Center for Biological Sequence Analysis, Department of Systems Biology, Technical University of Denmark, Kongens Lyngby, Denmark; 2 Department of Applied Mathematics and Computer Science, Technical University of Denmark, Kongens Lyngby, Denmark; 3 Department of Systems Biology, Technical University of Denmark, Kongens Lyngby, Denmark; Harvard Medical School, United States of America

## Abstract

Appearance is known to influence social interactions, which in turn could potentially influence personality development. In this study we focus on discovering the relationship between self-reported personality traits, first impressions and facial characteristics. The results reveal that several personality traits can be read above chance from a face, and that facial features influence first impressions. Despite the former, our prediction model fails to reliably infer personality traits from either facial features or first impressions. First impressions, however, could be inferred more reliably from facial features. We have generated artificial, extreme faces visualising the characteristics having an effect on first impressions for several traits. Conclusively, we find a relationship between first impressions, some personality traits and facial features and consolidate that people on average assess a given face in a highly similar manner.

## Introduction

We tend to evaluate others on their appearance and then move on to treat and interact with them based on these first impressions. Such an opinion can be formed after a tenth of a second from faces with neutral expressions and additionally people assess faces similarly for multiple traits, e.g. dominant and extraverted [1]–[3]. Specific facial features important for generating a first impression have been identified, for example is a large facial width-to-height ratio used as an indicator for a less trustworthy and more dominant personality type [4]–[6] - perhaps due to higher levels of testosterone in the blood resulting in a wider face [7]. Another important feature are the eyes; eye contact evokes trustworthiness [8]; and large eyes make a person appear more empathetic, agreeable, extraverted, conscientious and intelligent [9]. On one hand, there is some truth behind first impressions - it has been shown that valid inferences are made for at least four personality traits (Agreeableness, Conscientiousness, Extraversion, and Dominance) from facial features [10]–[13] - on the other hand, first impressions are not always accurate, e.g. people with infant-like facial traits (small chin, high eyebrows, and large eyes) are perceived as more emotionally warm, submissive, and naive [14], but often the direct opposite is true, as seen in many adolescent boys [15]. One cause of these inaccuracies is that people generate trait evaluations based on neutral facial expressions resembling actual emotional expressions – an effect named the overgeneralisation hypothesis [16].

To further delve into the generation and validity of first impressions, the differences and commonalities between faces have been studied extensively. An often-used approach is to go from a high-dimensional representation of a face, e.g. pixel values, 3-dimensional scans of faces or annotations of facial landmarks, to a lower-dimensional face-space by a Principal Component Analysis (e.g. [17]). Each dimension of the new face-space defines global properties of a face, which cannot be reduced to single features [3]. The implementation of such a face-space has made it possible to generate artificial faces supposedly expressing traits to a low or to a high degree [18], [19]. Walker and Vetter [20] used this technique to manipulate photographs of real faces making them appear more extreme for a given trait. Validation of these changed faces showed them to be chosen slightly more often than their non-extreme counterparts. In 2011 Rojas *et al.* showed facial trait evaluations as predicted automatically from facial features with high accuracy, revealing the consensus between participants when rating a face [21].

Since trait evaluations are connected to a person's facial structure it was our focus to generate a more complete picture of the relationship between facial features, trait evaluations made by others and measured personality traits. Our results confirmed the importance of facial features for trait evaluations and additionally some interesting connections between self-measured personality traits and first impressions surfaced. The artificial faces visualising the extremes of all traits were generated for men and women separately.

## Materials and Methods

### Ethics Statement

Data collection and analysis was performed in accordance with the Act of Processing of Personal Data and approved by the Danish Data Protection Agency before the beginning of the project. The participants were asked to give verbal informed consent to participate in this study and no data was collected until this consent was given. The consent is thereby documented by the recording of the data. This was in accordance to the guidelines of the Danish National Ethics Committee which state that written consent is only required if biological samples are collected, which was not the case in this study.

### Participants

Participants (N = 244, 128 women, 116 men) were recruited on campus at the Technical University of Denmark. All were either employed or studying at the university and between 18 years and 37 years old (μ = 24.56, σ = 3.24).

### Photographs

Facial photographs of all participants were taken with a Canon PowerShot XC200 camera under standardised conditions; controlled lighting, a white background and the same distance to the camera.

### Questionnaire

Each participant was instructed to fill out an online, Danish questionnaire composed of twelve questions regarding specific traits for twenty other randomly chosen, unacquainted participants from the cohort. Nine of the twelve traits were chosen to cover the personality traits measured with a self-report questionnaire and additionally we added the traits attractiveness, masculinity and physical health due to their possible effect on the other trait evaluations. The questionnaire was set up as a 9-range Likert scale with a neutral answer corresponding to five. The participants were instructed to evaluate each face for the traits friendly, adventurous, temperamental, physically healthy, extravert, dominant, attractive, masculine, emotionally stable, responsible and intelligent. The questions were phrased as “How [trait] does this person look” with the response scale ranging from 1, “Not [trait] at all”, to 9, “Very [trait]”. There was no time constraint for answering the questions and the faces were presented in randomised order. Approximately twenty participants rated each participant and the mean of the scores for each question was used as the actual score for that participant. Calculation of the Cronbach's α confirmed the reliability of this approach. The scores are further on referred to as the *Ratings*.

### Personality measurements

Cubiks In-depth Personality Questionnaire, CIPQ 2.0, a normative self-report questionnaire scoring 17 personality traits covering the Big Five [22] was used to measure the participants' personality traits. The personality traits were scored in a range from 1 to 10 and the results were assessed during a 45-minute session with a certified CIPQ test-scorer and the respondent. The test measures Neuroticism as its low pole: Emotional Stability. The scores are reversed compared to Neuroticism and additionally Emotional Stability focuses less on a person's level of anxiety and stress, but more on how emotionally perceptive and sensitive a person is. 226 participants completed the questionnaire.

### Appearance Model, AM

**Figure 1:**
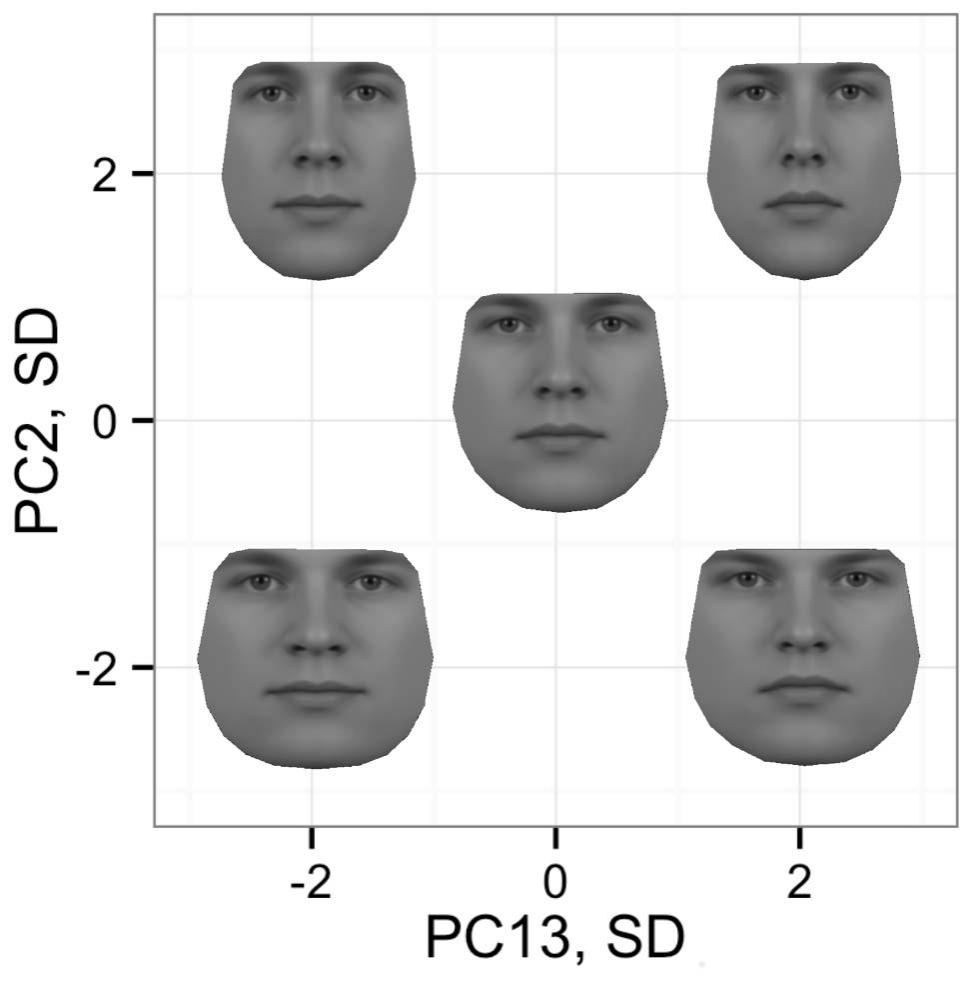
Example of two facial features, PC2 and PC13, and their interaction. The faces visualise how two principal components, PC, extracted by an Appearance Model, interact with each other. The coordinate system shows the change in a face when a principal component is moved two standard deviations in either the positive or the negative direction. The face in the middle shows the mean for all factors. E.g. the face in the upper right shows PC2 and PC13 at +2 standard deviations. It is seen that PC13 explains the shape of the mouth and PC2 the face width.

An Appearance Model, AM, which models all texture and shape information inside the boundaries of a face, was used to derive the facial components. Two models were generated, one for each gender, due to large differences in facial composition between men and women. The model is built by first annotating all photographs regarding the position and size of facial landmarks. Shape variations of the faces are extracted by a Principal Component Analysis, which in this case resulted in over 30 principal components each interpreting certain holistic facial characteristics of the participants (32 principal components for the male faces and 35 for the female faces). Afterwards the texture information is extracted through removing all shape information by warping all the images onto a mean shape. A Principal Component Analysis is performed on the pixel intensities in this set of shape-neutral images to model the variation in texture. This resulted in over 60 principal components explaining the texture of the faces (62 components for the male faces and 71 for the female faces). The resulting model contains a number of facial components describing a given face [23]. An example of two facial components and their interaction is shown in Figure 1.

### Data processing

Calculations were performed in R [24] and figures generated with the packages ggplot2 [25], pheatmap [26] and Cytoscape version 2.8 [27]. Differences in scores were tested for statistical significance with a Welch's t-test and the correlations between the individual *Ratings* and the self-measured personality traits were evaluated using the Pearson Correlation Coefficient, *r*. The significance of the correlations between the *Ratings* and the personality traits were confirmed by a permutation test, a statistical significance test, with 10,000 repeats. A permutation test repeatedly calculates correlations for randomised data to thereby find a measure for the significance of the actual correlation. A number of different models, both non-linear and linear, with varying subsets of facial features as predictors, were built for the prediction of the *Ratings* and the personality traits. The training was run as a 20-fold cross-validation and repeated thirty times for reliable standard deviations. Each model was trained on the training set using a growing number of the most correlated facial features (selected on the training set) as input. The Pearson Correlation Coefficient, *r*, between the observed and predicted Rating scores in the test set was used to evaluate the performance of each model. The best model was subsequently chosen based on its average performance on all folds.

### Extreme faces

The β-coefficients for the relevant predictors from the linear regression model were used to generate two extreme faces for each gender and each Rating. High and low scoring faces were calculated by applying four standard deviations to either the positive or the negative direction of the facial features. This was done to simulate faces evaluated as belonging to the ends of the Rating scale.

### Validation

The artificial, extreme faces were validated by asking 116 people, who were not part of the previous cohort, to choose from a set of four faces, which face they found to express a given personality trait the most. One of the four faces was the extreme face for the given Rating and the other three were randomly generated from the same parameter space. The validation was done as part of an open house at the Technical University of Denmark and therefore time constraints required us to only validate five traits for each gender. The traits were chosen to cover various aspects of the twelve *Ratings*. See Figure S4 for an example of a set of four images used in the validation.

## Results

The Cronbach's α ranged from 0.63 to 0.92 for the *Ratings* for each gender, with only *Responsible* and *Emotionally Stable* for the female faces having values below 0.70. Agreement between participants was generally larger for the male faces (0.80<α<0.92) than for the female faces (0.63<α<0.87). We used the average score for each *Rating* as a more reliable measure, based on responses from multiple people, for how a face is assessed by others.

**Figure 2:**
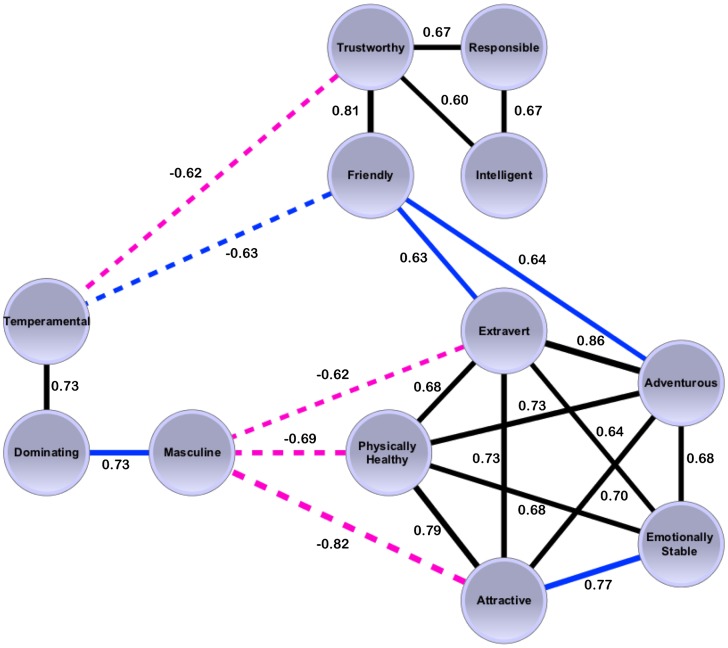
Network graph of all significant correlations between *Ratings*. The network depicts the relationship between the individual *Ratings* as the correlation coefficient, r, between scores. A dashed line depicts negative and a solid line positive correlations and the thickness of the line indicates the strength of the relationship with r as the edge label. Relationships significant for both genders are black, for men blue and for women magenta. Three clusters can be seen in the network with *Trustworthy, Responsible, Friendly* and *Intelligent* in the first, *Extraverted*, *Adventurous, Emotionally Stable, Attractive* and *Physically Healthy* in the second and *Temperamental, Dominating and Masculine* in the third. We named the clusters trustworthiness-friendliness, attractiveness-health-extraversion and dominance-masculinity.

The *Ratings* were seen to fall into three clusters (Figure 2), which we named dominance-masculinity, attractiveness-health-extraversion, and trustworthiness-friendliness. The attractiveness cluster seemingly represents the halo effect (the hypothesis stating that attractive people are evaluated more positively regarding positively loaded personality traits [28], [29]): High scores for Attractive clustered with high scores for Extraverted, Emotionally Stable, Physically Healthy, and Adventurous. We further discovered a clear link between scores for Dominating and Masculine for men (r(114) = .73, p<.001), which was in agreement with previous results [14], [30]. We compared the *Ratings* between genders with a Welsh's t-test and found that women generally are perceived as more trustworthy (p = 3.19×10^−5^), responsible (p = 4.40×10^−10^) and attractive (p = 6.35×10^−9^), whereas men are seen as more emotionally stable (p = 4.04×10^−6^).

**Figure 3:**
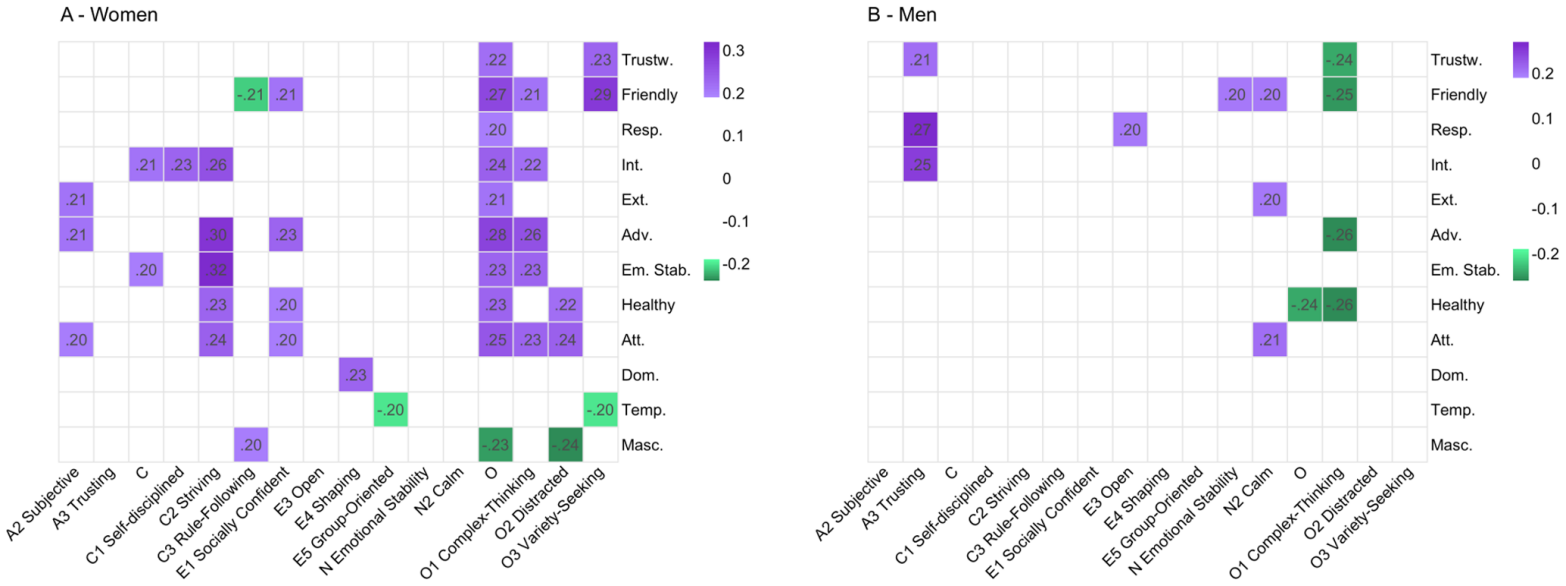
Correlations between *Ratings* and self-reported personality traits visualised by heat maps. Heat map A shows the correlations for women and heat map B the correlations for men. The personality traits are on the x-axis and the *Ratings* on the y-axis and a positive correlation is indicated with purple and a negative with green, where darker colours stand for bigger effect sizes. Only significant correlations with *abs(r)* ≥.20 and *p*<.01 are shown. Calculating the average of the correlations between personality traits and *Ratings* given by individual judges resulted in a drop in effect size; therefore the correlations in these heat maps should not be seen as significant on the individual level. Abbreviations for the *Ratings* are: Trustw.  =  Trustworthy, Adv.  =  Adventurous, Temp.  =  Temperamental, Healthy  =  Physically Healthy, Ext.  =  Extraverted, Dom.  =  Dominating, Att.  =  Attractive, Masc.  =  Masculine, Em. Stab.  =  Emotionally Stable, Resp.  =  Responsible and Int.  =  Intelligent.

Connecting the participants' personality-trait scores to the *Ratings* for each gender revealed subtle, but significant correlations (.20≤ *abs(r)* ≥.32, p<.01), which did not overlap between genders (see Figure 3). For men the most significant link was between evaluations for *Responsible* and the personality trait *Trusting*, a sub-trait of *Agreeableness* (*r*(116) = .27). Additionally we found a tendency that men with a more calm personality appear more friendly and extraverted (*r*(116) = .20). For women the strongest link was between the evaluations for *Emotionally Stable* and the personality trait *Striving*, a sub-trait of *Conscientiousness* (*r*(128) = .32). Dominance was also for women linked to higher scores in the corresponding personality trait Shaping (r(128) = .23, p<.01). Higher scores for *Openness to Experience* followed higher evaluations for many *Ratings* including the traits *Adventurous* (*r*(128) = .28) and *Friendly* (*r*(128) = .27). We found no connection between participants self-reported personality traits and the scores they gave others in the *Ratings*.

Since effect sizes from correlated average scores can be inflated [31], [32], we also correlated the raw scores given by each individual judge with the personality scores and then calculated averages and standard deviations for all these correlations based on individual judges. This resulted in effect sizes dropping below statistical significance (see Figure S1) with large standard deviations (.31<σ<.39) revealing a substantial individual factor in trait evaluations. Since it was our goal to investigate subtle effects of facial features on trait evaluations and we wanted a more complete measure of the trait evaluations we continued with the averaged scores, but the above found group-based effect sizes should be noted as inflated on the individual level.

Next, we explored the possibility of predicting single personality traits either from a person's *Ratings* or from his or her facial features. However, diverse non-linear approaches and varying subsets of predictors could not predict the personality traits, revealing the correlations as not strong enough for a stable prediction. The performance, when comparing the predicted and the observed personality traits, was low (r<.20, RMSE>2.00) and residual plots showed no satisfying fit.

**Figure 4:**
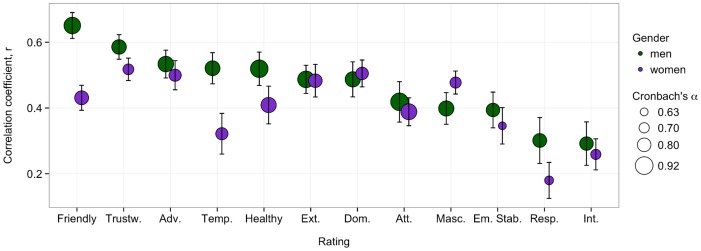
Prediction of *Ratings* from facial features. The plot shows the average correlation coefficient and standard deviation between observed and predicted scores for each *Rating* and each gender. A linear regression model was built in a 20-fold cross-validation with a varying number of the most correlated facial components as predictors, chosen based on the training set. Standard deviations are gathered by running the calculations thirty times with different folds for each run. The Ratings are in the plot ordered based on performance for the male faces. The size of the points indicates the Cronbach's α for that trait and it is seen that larger α-values correlate positively with prediction performance. Abbreviations for the *Ratings* are: Trustw.  =  Trustworthy, Adv.  =  Adventurous, Temp.  =  Temperamental, Healthy  =  Physically Healthy, Ext.  =  Extraverted, Dom.  =  Dominating, Att.  =  Attractive, Masc.  =  Masculine, Em. Stab.  =  Emotionally Stable, Resp.  =  Responsible and Int.  =  Intelligent.

The prediction of a person's *Ratings* from his or her facial features, however, gave more reliable results: it is to a certain extent possible to predict how a given person will be perceived based on his or her facial characteristics. We found a linear regression model to be most accurate, whereas more complex models (e.g. support vector machines with linear and radial kernels and a neural network with varying numbers of hidden nodes) did not improve the prediction significantly. The scores for Friendly for men were predicted with the highest accuracy (*r* = .65, *σ* = 0.04). Figure 4 visualises the correlation between observed and predicted scores for all *Ratings* for both genders with the corresponding Cronbach's α We observed predictions being overall better for male faces (p<.001), which is in agreement with the higher values of Cronbach's α for these. The correlation between the Cronbach's α and the prediction accuracy was substantial (*r* = .51, p<.02), again confirming the importance of the agreement between raters for the validity of a given prediction [20].

**Figure 5:**
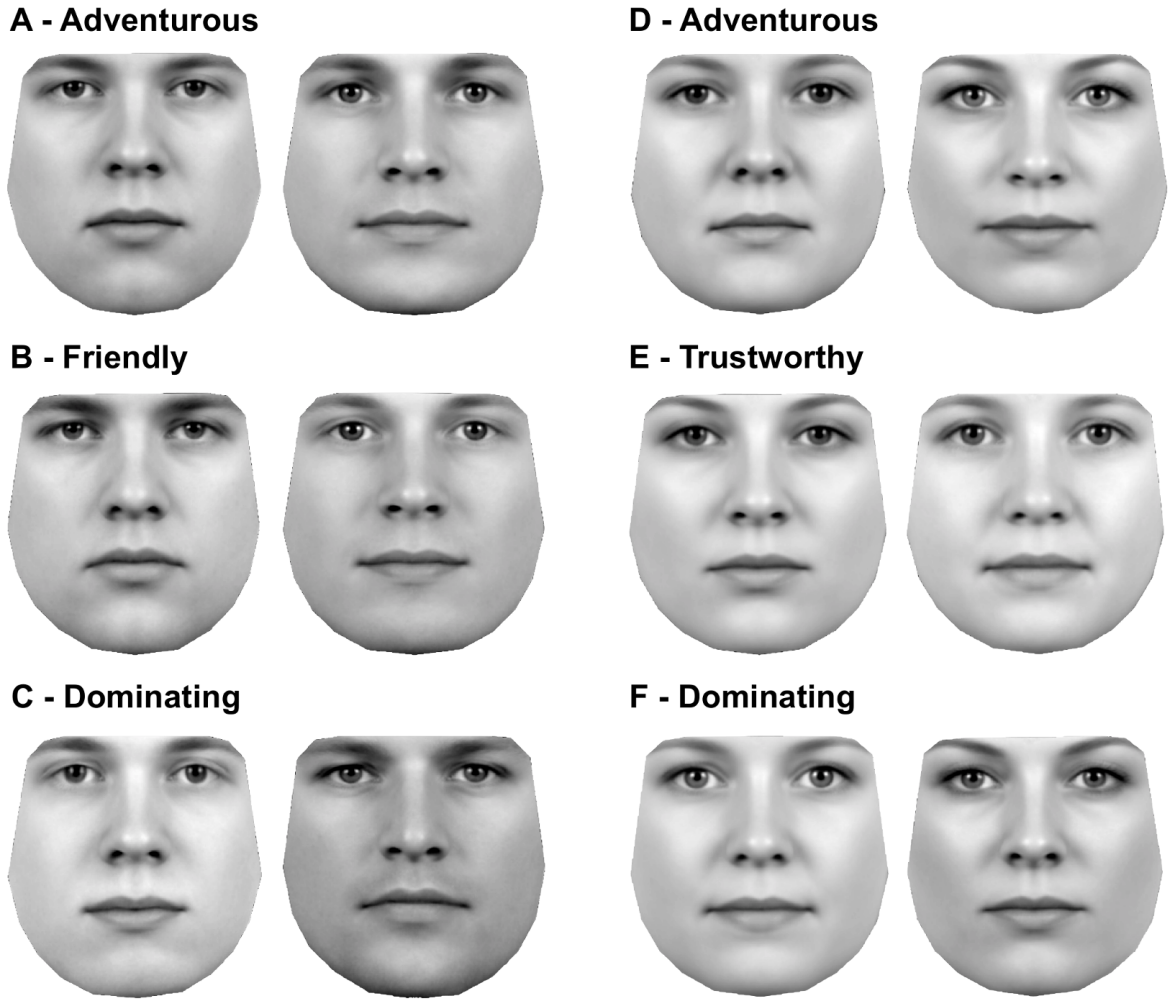
Extreme faces for the *Ratings*. For each face pair the left extreme face is predicted as being judged very low for a given trait and the right face as very high. Each face is based on the β-coefficients from the best linear regression model for that given Rating and gender. We generated the faces by multiplying each β-coefficient to either +4 standard deviations or -4 standard deviations of the matching facial component. A: Male extremes for *Adventurous*. B: Male extremes for *Friendly*. C: Male extremes for *Dominating*. D: Female extremes for *Adventurous*. E) Female extremes for *Trustworthy*. F: Female extremes for *Dominating*.

To visualise the models we generated artificial faces predicted to express a given trait either to a high or a low degree. Three pairs of these extreme faces for each gender are shown in Figure 5 and all face pairs are shown in Figures S2 and S3. Our model is built from holistic features and therefore it is difficult to conclusively state much about specific parts of a face, but some differences stand out in the extreme pairs. For appearing friendly the mouth seems to have an impact: a wider mouth with neutrally or upwards pointed corners of the lips resulted in higher scores for friendliness (Figure 4B). The male extreme faces for Dominating (Figure 4C) reveal the effect of a wider face and a more pronounced eyebrow-ridge. For women the extreme faces for Adventurous (Figure 4D) indicate a positive impact of fuller lips and dark lashes (possibly eye make-up).

**Figure 6:**
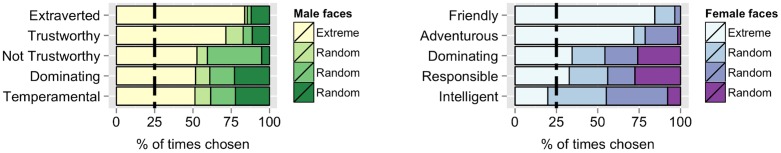
Validation of extreme faces. In the validation we presented four faces to 116 persons and asked them to choose which one they found to represent a given trait the most. The left plot shows results for the male extremes and the right results for the female extremes. The length of each section in each bar indicates the percentage of times the given face was chosen. The dotted line indicates the percentage representing a random selection of the extreme face. In all cases except one the extreme face was chosen more often than random. For the male faces we found the extremes to be chosen significantly over random. For the female faces this was only found for the *Friendly* and *Adventurous* extremes. The colours are from [33].

We performed a validation of our extreme faces by asking 116 people outside the original study to choose between four artificial faces the one that looked to posses a certain personality trait to the highest degree; one of the four faces was the extreme face for the given trait. Due to time constraints this was done for only five traits for each gender and we saw that the extreme face was chosen above random in all cases except one, Intelligent for women. This unsuccessful case could be connected to a lower agreement between raters when evaluating this trait (α = 0.70). In Figure 6 we show the percentage of times each face was chosen for each question and how this compares to a random selection between the four faces. Validation of the male extreme faces was successful: participants selected the extreme male face significantly more often than the random faces for the five traits (p<.001), which fits the fact that the prediction of the *Ratings* for the male faces had an overall good performance and the higher agreement between raters for the male faces. Validation of the female extreme faces was significant for only two of the five chosen traits, Friendly and Adventurous (p<.03). The other three extremes for the traits Dominating, Responsible and Intelligent were not chosen significantly more often than the random faces, which is in concordance with the lower prediction performance, especially for *Responsible* and *Intelligent*.

## Discussion

We found the prediction of personality traits from facial features to be unsuccessful, but we discovered that some traits could be inferred from a face to a certain extent. The identified connections between individual personality traits and *Ratings* were subtle but significant and mostly in accordance with previous research. For women we confirmed that inferences could be made about the level of *Openness*, *Striving* and *Dominance* from a face. For men a calmer personality linked with higher evaluations for friendliness and a more trusting personality with higher scores for responsibility. The latter could indicate that a person's level of trust in others can be influenced by his or her appearance, if appearance makes others treat him or her as more responsible. Some of these correlations have been reported previously [10], [11], [13], but seldom from only facial photographs as input [12], [34].

The above results were based on average *Ratings*; correlations for *Ratings* given by individual judges were significantly smaller and in some cases shrunk down to zero. Thus, when assessing individual scores for a connection between trait evaluations and personality, an effect does not seem apparent: none of the average correlations on the individual level were statistically significant. We have two arguments for why this happens. Firstly, the individual correlations are only based on responses for about ten faces, since each judge rated approximately ten people of each gender, which leads to a much higher uncertainty in the correlation. Secondly, if there is a real connection between a trait and facial appearance, then based on classical test theory the averaging of several scores can reduce the trait evaluation error. This happens because each score is composed of a true component and an error component, leading to a decrease in error when scores from several raters are combined. Consequently we still see the average *Rating* score as reliable for assessing trait evaluations from facial features, although the found correlations between personality traits and trait evaluations should be seen as only valid on a group-based level.

As others, we confirmed that people evaluate faces similarly for several traits, which manifested itself in a fairly accurate prediction of how people perceive a face based on facial features (e.g. [1]). This effect was supported by a validation of our generated extreme faces. The validation success was seen to be somewhat dependent on agreement between raters: traits with higher Cronbach's α were generally predicted with higher accuracy. For some traits, e.g. *Responsible* and *Intelligent* for women, the reliability of judgments was low, which revealed these traits as subjectively evaluated. In general raters agreed more on how to evaluate male faces.

The extreme faces confirmed the impact of a larger facial width-to-height ratio for appearing more dominating [4]. The shape of the mouth was also seen to have an impact, with neutral or upwards pointed corners of the lips resulting in higher scores for positively loaded traits. This could specifically be due to the overgeneralisation hypothesis leading to false trait judgments, since a more smiling expression connects well with emotional expressions for positive traits [16], [20].

The *Ratings* were seen to fall into three clusters, dominance-masculinity, trustworthiness-friendliness, and attractiveness-health-extraversion. These three clusters fit previous findings showing three factors as sufficient for evaluating a face. Two of these factors, valence/trustworthiness and dominance, were discovered by Oosterhof and Todorov [18] and confirmed by Sutherland *et al*. [35]. Sutherland *et al*. additionally detected a youthful-attractiveness factor, which connects to our attractiveness-health-extraversion cluster. These studies also found emotional expressions like anger and happiness to correlate strongly with trust evaluations, an effect also apparent in our extreme faces.

Perhaps the fact that faces are assessed based on the overgeneralisation hypothesis led to us not finding a clear relationship between trait evaluations and self-measured personality scores. Studies using short video sequences of a person instead of a photograph have reported more precise first impressions [36], strengthening the belief of a connection between personality and appearance. It seems that a single facial photograph lacks information for evaluating diverse traits: a viewer will miss additional cues for gathering a more complete first impression from a face and will therefore instead focus overly on facial expressions.

In conclusion our results confirm the impact facial features have on first impressions and that people generally agree on how to evaluate some aspects of personality based on a face, even though these evaluations often are far from the self-measured personality traits. We replicated previous findings about three factors being sufficient for trait evaluations. We believe that appearance has an impact on personality development, since social interactions are such a monumental part of our lives. A slight indication of this was also found in some of the connections between self-reported personality traits and trait evaluations and in studies involving video sequences of ratees, but more research is at the moment needed to prove the directionality and size of this effect.

## Supporting Information

Figure S1 **Heat maps for the averaged correlations between** ***Ratings*** **given by individual judges and the self-reported personality traits.** Heat map A shows the correlations for women and heat map B the correlations for men as a 95% confidence interval. The personality traits are on the x-axis and the *Ratings* on the y-axis and a positive correlation is indicated with purple and a negative with green, where darker colours stand for bigger effect sizes. Only the correlations significant in the correlated averages in Figure 3 are shown and a large drop in effect size is seen compared to these. Abbreviations for the *Ratings* are: Trustw.  =  Trustworthy, Adv.  =  Adventurous, Temp.  =  Temperamental, Healthy  =  Physically Healthy, Ext.  =  Extraverted, Dom.  =  Dominating, Att.  =  Attractive, Masc.  =  Masculine, Em. Stab.  =  Emotionally Stable, Resp.  =  Responsible and Int.  =  Intelligent.
(TIFF)

Figure S2 **Male extremes for the** ***Ratings*** **.** The extreme face scoring low for a given trait is depicted on the left and the extreme face scoring high on the right for each *Rating*. The traits are ordered based on prediction performance.
(TIFF)

Figure S3 **Female extremes for the** ***Ratings*** **.** The extreme face scoring low for a given trait is depicted on the left and the extreme face scoring high on the right for each *Rating*. The traits are ordered based on the prediction performance for the male faces.
(TIFF)

Figure S4 **Example of a validation question,** ***Intelligent*** **.** The upper left face is the generated extreme for the trait *Intelligent*. The other three are randomly generated from the same parameter space as the extreme face. The extreme face for *Intelligent* was the only one that was not chosen over random in the validation, which matched the fact that *Intelligent* also was predicted with the lowest performance.
(TIFF)

File S1 **Supporting Information.** Table S1, questions used for the *Ratings*. Table S2, participant information. Table S3, scores given in the *Ratings*. Table S4, PCA scores for women with participants in columns. Table S5, PCA scores for men with participants in columns. Table S6, validation questions. Table S7, validation participant information. Table S8, scores given in the validation.
(ZIP)
