# Supplementary figures and images for: Interpretation of Appearance: The Effect of Facial Features on First Impressions and Personality
Source: PLoS One. 2014 Sep 18;9(9):e107721. doi: 10.1371/journal.pone.0107721 (PMC4169442; doi:10.1371/journal.pone.0107721)

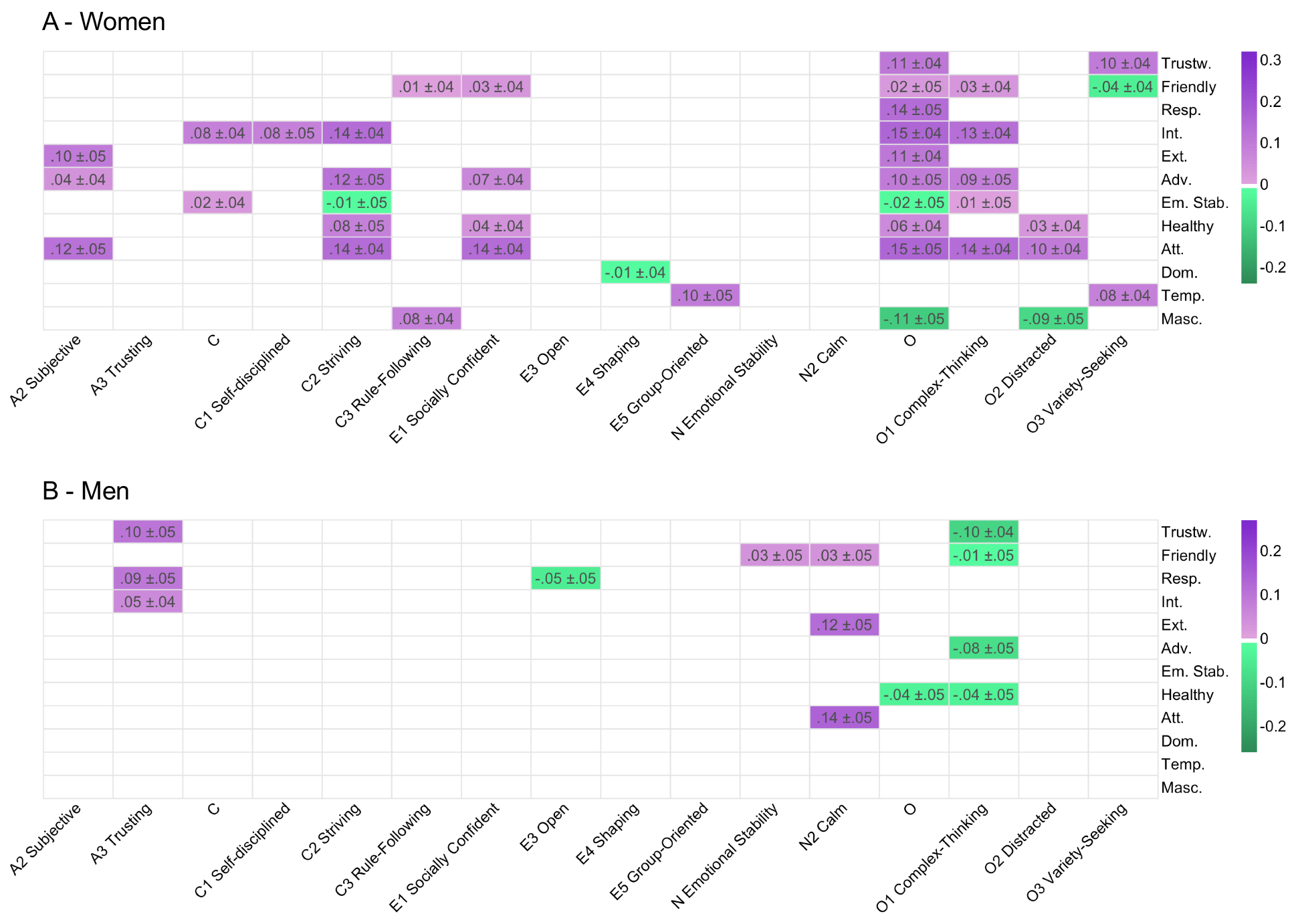

Supplement: Figure S1 — Heat maps for the averaged correlations between Ratings given by individual judges and the self-reported personality traits. Heat map A shows the correlations for women and heat map B the correlations for men as a 95% confidence interval. The personality traits are on the x-axis and the Ratings on the y-axis and a positive correlation is indicated with purple and a negative with green, where darker colours stand for bigger effect sizes. Only the correlations significant in the correlated averages in Figure 3 are shown and a large drop in effect size is seen compared to these. Abbreviations for the Ratings are: Trustw. = Trustworthy, Adv. = Adventurous, Temp. = Temperamental, Healthy = Physically Healthy, Ext. = Extraverted, Dom. = Dominating, Att. = Attractive, Masc. = Masculine, Em. Stab. = Emotionally Stable, Resp. = Responsible and Int. = Intelligent. (TIFF) [file pone.0107721.s001.tiff]

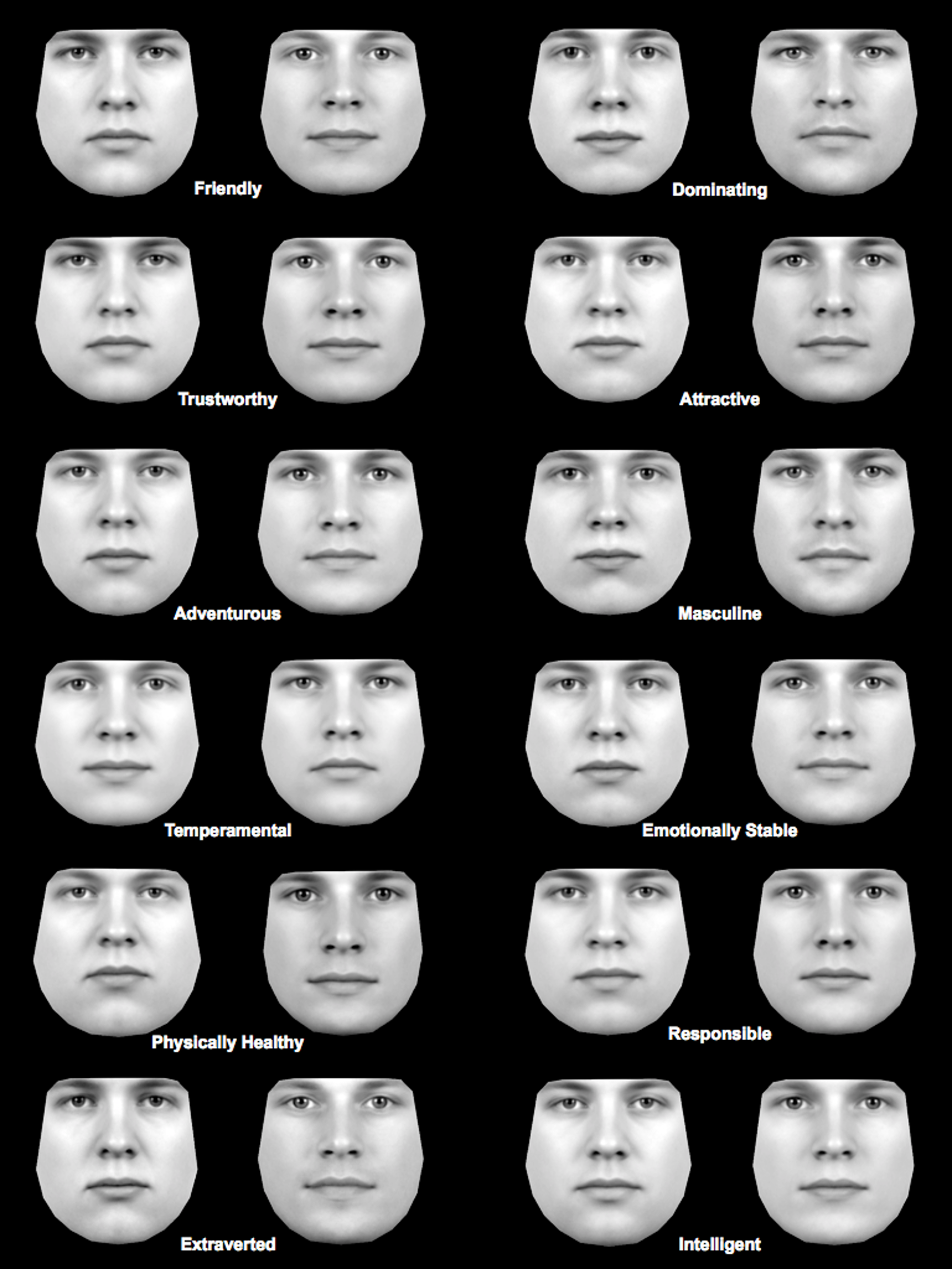

Supplement: Figure S2 — Male extremes for the Ratings . The extreme face scoring low for a given trait is depicted on the left and the extreme face scoring high on the right for each Rating. The traits are ordered based on prediction performance. (TIFF) [file pone.0107721.s002.tiff]

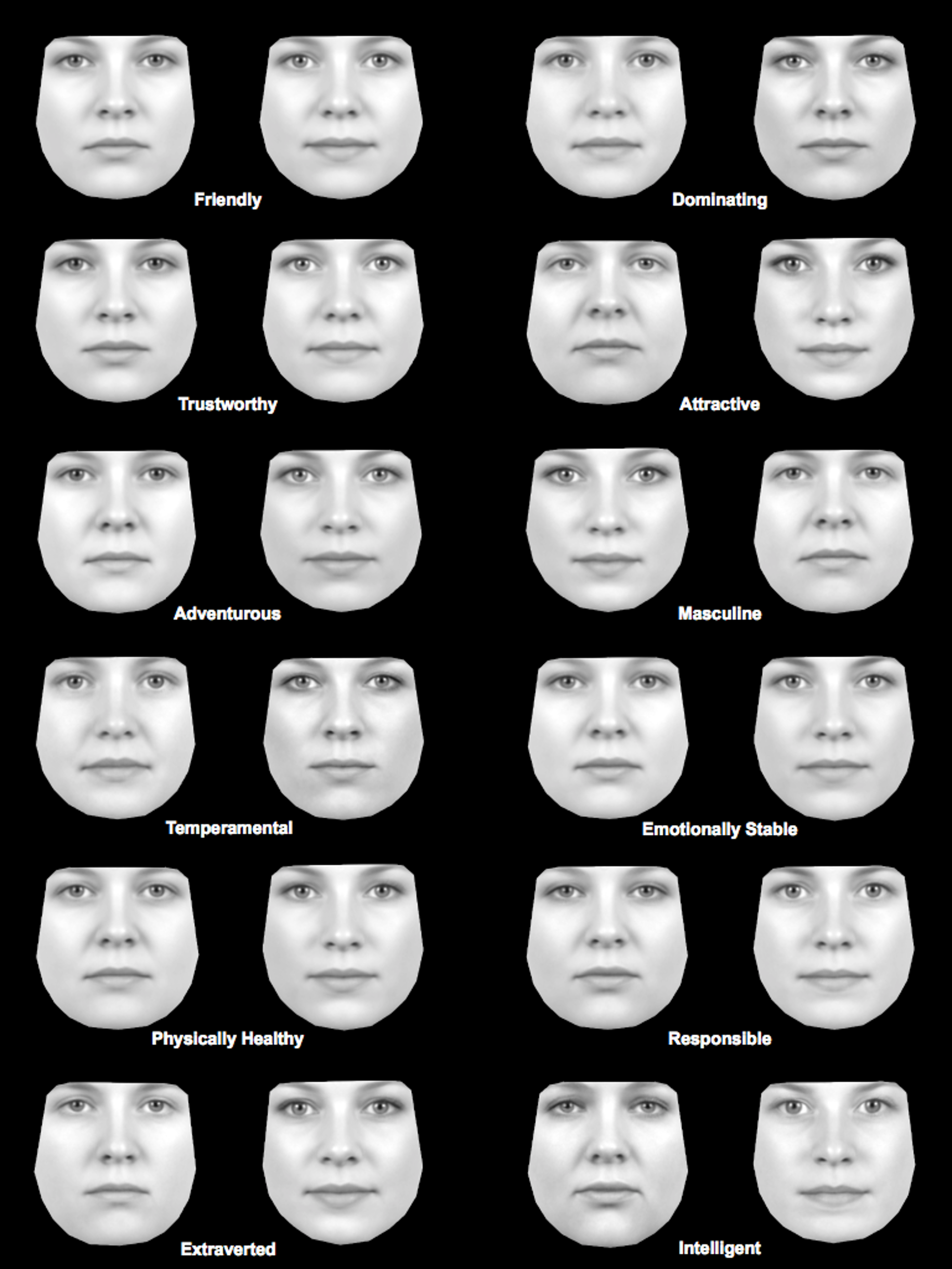

Supplement: Figure S3 — Female extremes for the Ratings . The extreme face scoring low for a given trait is depicted on the left and the extreme face scoring high on the right for each Rating. The traits are ordered based on the prediction performance for the male faces. (TIFF) [file pone.0107721.s003.tiff]

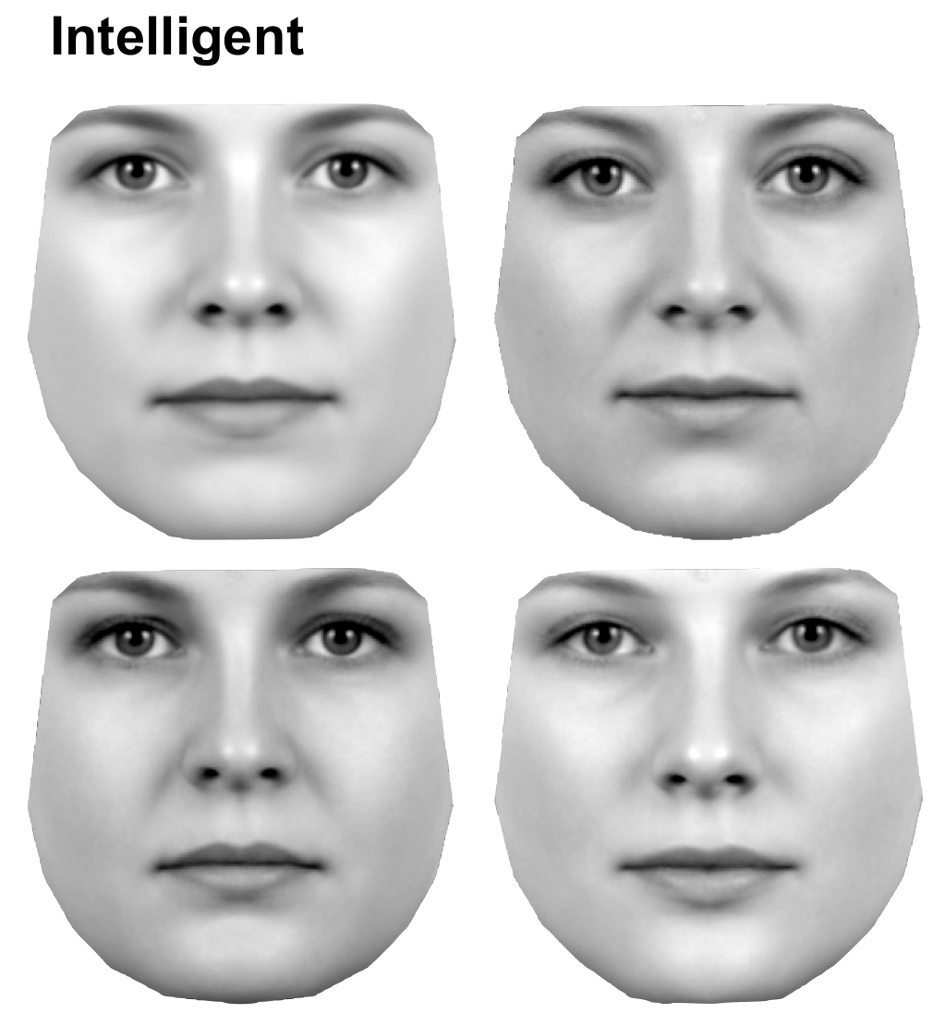

Supplement: Figure S4 — Example of a validation question, Intelligent . The upper left face is the generated extreme for the trait Intelligent. The other three are randomly generated from the same parameter space as the extreme face. The extreme face for Intelligent was the only one that was not chosen over random in the validation, which matched the fact that Intelligent also was predicted with the lowest performance. (TIFF) [file pone.0107721.s004.tiff]
